# Host Longevity and Parasite Species Richness in Mammals

**DOI:** 10.1371/journal.pone.0042190

**Published:** 2012-08-06

**Authors:** Natalie Cooper, Jason M. Kamilar, Charles L. Nunn

**Affiliations:** 1 Department of Human Evolutionary Biology, Harvard University, Cambridge, Massachusetts, United States of America; 2 School of Natural Sciences, Trinity College Dublin, Dublin, Ireland; 3 Trinity Centre for Biodiversity Research, Trinity College Dublin, Dublin, Ireland; 4 Department of Anatomy, Midwestern University, Glendale, Arizona, United States of America; 5 School of Human Evolution and Social Change, Arizona State University, Tempe, Arizona, United States of America; University of Utah, United States of America

## Abstract

Hosts and parasites co-evolve, with each lineage exerting selective pressures on the other. Thus, parasites may influence host life-history characteristics, such as longevity, and simultaneously host life-history may influence parasite diversity. If parasite burden causes increased mortality, we expect a negative association between host longevity and parasite species richness. Alternatively, if long-lived species represent a more stable environment for parasite establishment, host longevity and parasite species richness may show a positive association. We tested these two opposing predictions in carnivores, primates and terrestrial ungulates using phylogenetic comparative methods and controlling for the potentially confounding effects of sampling effort and body mass. We also tested whether increased host longevity is associated with increased immunity, using white blood cell counts as a proxy for immune investment. Our analyses revealed weak relationships between parasite species richness and longevity. We found a significant negative relationship between longevity and parasite species richness for ungulates, but no significant associations in carnivores or primates. We also found no evidence for a relationship between immune investment and host longevity in any of our three groups. Our results suggest that greater parasite burden is linked to higher host mortality in ungulates. Thus, shorter-lived ungulates may be more vulnerable to disease outbreaks, which has implications for ungulate conservation, and may be applicable to other short-lived mammals.

## Introduction

Understanding parasite infections in wild animals is of great importance. For example, infectious diseases are threatening various species (e.g., amphibians and Tasmanian devils; [1], [2]), while biodiversity itself may influence the prevalence of parasites in ecological communities [3], [4]. Additionally, we share approximately 60% of our infectious diseases with animals [5] and many recent human pandemics originated in wildlife, including HIV and SARS [6], [7]. Identifying the host characteristics that support multiple parasites is therefore critically important for human health and the conservation of biodiversity.

Mammals are infected by a wide variety of parasites, ranging from microscopic viruses and bacteria to macroscopic tapeworms, flukes and biting arthropods [4], [8]. These parasites are also diverse in terms of their transmission modes (e.g., sexual, vertical, vector-borne, airborne, and fecal-oral) and life cycles (e.g., direct or via one or more intermediate hosts). The diseases caused by these infectious agents can have profound fitness effects on individual hosts, resulting in selection for anti-parasite behaviors [9], immune defenses [10], and changes in life-history features such as birth weight [11]. Despite a great deal of study, however, it remains unclear how parasites influence many aspects of host biology, including basic life-history parameters.

Host longevity is a life-history parameter that is expected to covary with parasite infection [12]. A number of comparative studies have investigated the relationship between these variables in mammals but results have been mixed; some studies found limited evidence that longer-lived mammals had more parasites [13], some studies found that longer-lived mammals had fewer parasites [12], [14], while other authors failed to find evidence for an association [15]–[18]. Additionally the relationship between parasites and host longevity is unclear because causality may be bidirectional, with parasites influencing measures of longevity, while longevity simultaneously influences parasite success. We describe these two competing hypotheses below using parasite species richness as our measure of parasite burden.

Parasites often cause negative fitness effects on hosts and, while some behavioral defenses may help species avoid infection, a substantial level of unavoidable infection (and therefore mortality) is likely to exist in wild populations [12]. Thus, similar to the effects of unavoidable mortality through predation, higher parasite pressure may favor a shorter lifespan (and faster reproduction). This should result in a negative correlation between parasite burden and host longevity, with higher parasite species richness in shorter-lived species.

Conversely, host longevity may influence parasite burden through epidemiological processes, predicting a positive association between longevity and parasite richness. Increases in host background mortality should make it more difficult for parasites to establish in host populations because the death of a host also results in the death of its parasites. Given that a higher background mortality rate is equivalent to a shorter longevity, it is reasonable to expect that more parasites will meet the conditions for establishment (i.e., R_0_>1; [19]) in hosts that live longer. Based on these basic epidemiological principles, we expect to find a positive correlation between parasite species richness and host longevity, with highest parasite species richness in long-lived species [20]. Increased longevity may also lead to greater parasite species richness because a longer-lived individual is likely to be exposed to more parasites throughout its lifetime [12], [21]. Although not all of these infections will be retained throughout the life of an individual, sampling across individuals should reveal more species of parasite in longer-lived host species.

Host immune investment may provide crucial insights into the relationship between host longevity and parasite burden. Immune investment is costly, so one might expect a trade-off between immune investment and investment in other life-history traits such as growth and reproduction [22]. A heavily parasitized host may achieve the same fitness by either (a) investing in immunity and reproducing over a longer lifespan, or (b) investing in rapid reproduction to the detriment of immune investment, leading to increased mortality and a shorter lifespan. Thus, immune investment may either decrease or increase with parasite burden. In addition, the optimal life-history strategy may depend on the kind of infections to which the host is exposed: chronic infections may select for increased immune investment and a longer lifespan, whereas acute infections with high mortality rates may select for a faster life-history, reduced immune investment and shorter longevities.

Here, we investigate the relationship between maximum longevity and parasite species richness in mammals using data from terrestrial Carnivora, Primates and terrestrial ungulates (Artiodactyla and Perissodactyla). Our study extends previous studies and aims to resolve previously conflicting findings by more than doubling the number of host species in the comparative dataset. Compared to previous research, we also use more advanced phylogenetic methods, including methods to estimate and take into account phylogenetic signal in the data, while rigorously controlling for the potentially confounding effects of body mass and sampling effort (for estimates of both parasite species richness and maximum longevity). We also investigate the relationships among immune system investment, maximum longevity and parasite species richness.

## Materials and Methods

### Data

We used parasite species richness (PSR) data from the *Global Mammal Parasite Database* (GMPD; [23]). This database contains host-parasite records taken from the literature since 1929, and continues to be updated as new papers are published. All records come from wild host populations and represent natural infections. To date, the database contains over 20,000 host-parasite records from over 500 host species and over 2100 parasite species, including both macro- (i.e., helminthes) and micro- (i.e., viruses, bacteria, protozoa and fungi) and ecto-parasites (i.e., arthropods). The GMPD contains information on parasites found in wild Carnivora, Primates and terrestrial ungulates (Artiodactyla and Perissodactyla); thus we restricted our analyses to these groups. We excluded the marine Carnivora (Phocidae, Otariidae, Odobenidae) because aquatic environments may result in differences among parasite transmission patterns, immune investment and life-history features (e.g., aquatic carnivores have higher white blood cell counts than terrestrial carnivores; [24]).

We estimated total parasite species richness (PSR) for each host species, using the taxonomy of Wilson and Reeder [25], and also estimated PSR for macro- (i.e., helminthes) and micro-parasites (i.e., viruses, bacteria, protozoa and fungi) separately (PSR_macro_ and PSR_micro_). For some host-parasite records, parasites were identified only to the genus-level. To use as much data as possible, we included these parasites in estimates of PSR provided that no other members of the genus were recorded for the host species. In total, our PSR values used 2174 species of parasite (994 macro-, 779 micro- and 401 ecto-parasites).

For each host species, we then collated data on maximum longevity (months) from the PanTHERIA and AnAge databases [26], [27], Walker’s Mammal Species of the World [28], and a few additional sources (Supporting Information S1). We used a mammal supertree for all phylogenetic analyses [29], [30].

Both PSR and longevity show correlations with body mass in some mammals (e.g., [13], [14], [31]–[33]). Thus, any correlation between longevity and PSR could be the result of covariation with body mass. To address this possibility, we included body mass in our models (see below). We collated data on adult body mass (g) from PanTHERIA and AnAge [26], [27], Walker’s Mammal Species of the World [28], and a few additional sources (Supporting Information S1). We note that other variables also covary with taxonomic subsets of PSR in some mammals, including social group size and geographic range size (e.g., [13]). However, when we performed phylogenetic generalized least squares models (see below) controlling for body mass and sampling effort, these variables were not correlated with PSR for carnivores or ungulates (Table S1). We found weak significant positive correlations between PSR and both social group size and geographic range size for primates (Table S1). However, these significant associations disappear in full models (Table S2). To simplify our results, we therefore do not include social group size or geographic range size in the statistical models investigated here.

PSR and life-history data are also sensitive to sampling effort: host species which have been thoroughly sampled for parasites may appear to have higher PSR values than those which have been less well-sampled [13], [34]. Similarly, a well-studied host species may appear to have higher maximum longevity than its less-well studied counterparts [33], [35]. To control for these sampling biases we included a measure of sampling effort (citation count) for each host species. We defined this as the number of ISI Web of Knowledge (http://wokinfo.com/) references where the Latin binomial of the species appeared in either the title or topic fields. Where the species binomial had changed between the 1993 and 2005 taxonomies [25], [36] we summed the number of citations for the species names from both taxonomies.

For analyses of host immune investment, we extracted mean white blood cell counts (WBC; expressed as the number of cells in 10^−9^ liters of blood) from the International Species Information System (ISIS) database [37]. We used WBC as a proxy for host immune investment because white blood cells represent the first line of defense against pathogens, they are probably costly to produce, and WBC is used by both physicians and wildlife ecologists to gauge the health of individuals (e.g. [38]). Within primates, for example, significantly higher WBC are observed in diseased individuals [39]. Other components of the vertebrate immune system, such as spleen size and the diversity of major histocompatibility complex (MHC) genes are also likely to be important indicators of immune investment; however, these data are not available for most of our species.

The ISIS database contains physiological data from putatively healthy captive individuals only. This helps to remove the confounding effects of differences in health or stress levels on physiology. Ideally, we would use data from wild individuals with information on their health and stress levels. However, these data are rare for wild populations making the ISIS database the best alternative available. Although WBC may vary between sexes and among age classes [40], most ISIS records do not separate WBC records into separate sexes or age classes for all species in our dataset. Thus, we used WBC from all ages and sexes combined to get the largest sample size possible.

In total we have data on PSR, longevity, body mass and citation counts for 361 species (132 carnivores, 128 primates and 101 ungulates). We also have white blood cell counts for 219 of these species (64 carnivores, 81 primates and 74 ungulates). The data are available in Supporting Information S2.

### Analyses

We found that natural-log transformed data improved model diagnostics, resulting in a better distribution of residuals from the regression model. Thus, all variables were ln-transformed prior to analysis. Before fitting multivariate models, we also checked the predictors for collinearity (following the method of [41]) because it can lead to unreliable model parameter estimates. Variance inflation factors (VIF) were less than three, indicating acceptable levels of collinearity [41].

Species in comparative analyses are related to one another and thus may share similarities because they inherited them from a common ancestor, rather than through independent evolution [42], [43]. To deal with the potential statistical non-independence of the interspecific data, we used phylogenetic generalized least squares models (PGLS). PGLS is based on the usual GLS model except that the phylogenetic dependence of the data is incorporated into structure of the error term [44]–[46]. This error term can be constructed in a number of ways. Here it consists of a matrix of expected trait covariances calculated using the phylogeny and the maximum likelihood (ML) estimate of λ. The parameter λ is a multiplier of the off-diagonal elements of a phylogenetic variance-covariance matrix that best fits the data, and varies between λ = 1, where the data are structured according to a Brownian motion model of trait evolution, and λ = 0, where the data show no phylogenetic structure and the analysis reduces down to a non-phylogenetic OLS analysis [45], [47]. For each regression, λ is estimated for the residual error term [48], along with the other regression parameters so regressions are carried out whilst controlling for the actual degree of phylogenetic non-independence present. For interest, we report the phylogenetic signal (λ) in individual variables in Table S3, however we note that this does not provide any justification for using PGLS or non-phylogenetic methods [48].

We used R v.2.13.0 [49] to run all of the analyses. Specifically, we used the function pgls in the package caper [50] to fit the following model for carnivores, primates and ungulates separately:


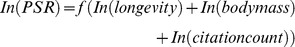
(1)

We focus on these three clades separately because each offers sufficient sample sizes to test the hypotheses, and when the data are combined, we found that patterns were driven by a strong positive relationship in only one of the clades (ungulates). To test relationships among longevity, immune system investment and parasite species richness, we also used PGLS to fit the following model for carnivores, primates and ungulates separately:


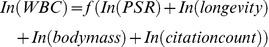
(2)

We predict that different types of parasites will affect host longevity in different ways; specifically we expect chronic infections to select for longer lifespans and increased immune investment, and acute infections to select for shorter lifespans and decreased immune investment. Therefore we also fitted each model using PSR for macro- and micro-parasites separately, because macroparasites are generally thought to cause chronic infections and microparasites to cause acute infections [51]. Obviously there are exceptions to this generalization; however, data on the type of infection was unavailable for most of our parasite species so this was the best approximation available.

The statistical performance of PGLS can be strongly influenced by outliers, especially where large evolutionary changes have occurred on short branches. This can result in points with very high leverage that could affect parameter estimates and increase the error rates of the regressions. To avoid this, we repeated our regressions after removing any points with a studentized residual exceeding ±3 [52]. However, results were qualitatively similar, and so we only report results from analyses in which all the data were used.

We also used phylogenetic analysis of variance (ANOVA) to investigate differences among our three host groups in their PSR, longevity, body mass, and WBC values. Phylogenetic ANOVAs perform a standard ANOVA but determine the significance value of the F statistic by comparing the observed value to a null distribution obtained by simulating new sets of data under a Brownian motion model along the phylogeny [53]. We fit these using the function phy.anova in the package geiger [54].

## Results

We found a significant negative relationship between maximum longevity and total parasite species richness (PSR) for ungulates but not for carnivores or primates (Table 1). When we investigated macro- and micro-parasites separately, only ungulates showed a significant negative relationship between maximum longevity and microparasite species richness (PSR_micro_; Table 1). The PSR values of the three mammalian groups were not statistically different, suggesting that variation in the results was not due simply to clade specific differences in total parasite burden, or differences in the numbers of macro- or micro-parasites infecting each clade (Table 2). However, we did find significant differences in longevity among groups; primates have the longest lifespans, followed by ungulates and carnivores (Table 2).

**Table 1:** Phylogenetic generalized least squares models (PGLS) predicting total parasite species richness (PSR), macroparasite species richness (PSR_macro_) or microparasite species richness (PSR_micro_) for Carnivora, Primates and terrestrial ungulates.

		**Carnivora**			**Primates**			**Ungulates**	
**PSR**	**λ = 0.056**	**r^2^ = 0.556**	**AIC = 367.2**	**λ<0.001**	**r^2^ = 0.454**	**AIC = 330.0**	**λ<0.001**	**r^2^ = 0.237**	**AIC = 345.4**
**variable**	**slope**	**SE**	**t_128_**	**slope**	**SE**	**t_124_**	**slope**	**SE**	**t_97_**
Longevity	−0.022	0.282	−0.079	−0.208	0.307	−0.676	−0.911	0.417	−2.184*
Body mass	−0.047	0.063	−0.743	0.060	0.074	0.817	0.077	0.123	0.627
Citations	0.835	0.071	11.70***	0.528	0.059	8.913***	0.526	0.099	5.318***
**PSR_macro_**	**λ = 0.234**	**r^2^ = 0.489**	**AIC = 353.9**	**λ = 0.619**	**r^2^ = 0.349**	**AIC = 307.8**	**λ <0.001**	**r^2^ = 0.168**	**AIC = 326.5**
**variable**	**slope**	**SE**	**t_128_**	**slope**	**SE**	**t_124_**	**slope**	**SE**	**t_97_**
Longevity	0.109	0.281	0.388	0.099	0.300	0.329	−0.535	0.380	−1.409
Body mass	−0.116	0.067	−1.737	0.064	0.098	0.654	−0.086	0.112	−0.767
Citations	0.698	0.068	10.22***	0.375	0.054	7.001***	0.398	0.090	4.420***
**PSR_micro_**	**λ = 0.062**	**r^2^ = 0.535**	**AIC = 287.2**	**λ = 0.325**	**r^2^ = 0.158**	**AIC = 307.4**	**λ<0.001**	**r^2^ = 0.453**	**AIC = 274.7**
**variable**	**slope**	**SE**	**t_128_**	**slope**	**SE**	**t_124_**	**slope**	**SE**	**t_97_**
Longevity	0.042	0.209	0.199	−0.080	0.286	−0.280	−0.744	0.294	−2.530*
Body mass	−0.017	0.047	−0.362	0.158	0.079	1.998	0.220	0.087	2.536*
Citations	0.577	0.053	10.94***	0.356	0.054	6.637***	0.541	0.070	7.759***

*** p<0.001;

** p<0.01;

* p<0.05.

**Table 2:** Results of phylogenetic ANOVAs testing for differences in variables among Carnivora, Primates and terrestrial ungulates.

Variable	df	F	phylo.p	details
PSR*	2, 327	3.779	0.331	NA
PSR_macro_ *	2, 211	0.449	0.819	NA
PSR_micro_ *	2, 211	1.474	0.441	NA
Longevity	2, 327	28.23	<0.001	Primates > Ungulates > Carnivora
Body mass	2, 327	158.7	<0.001	Ungulates > Carnivora > Primates
Citations	2, 327	0.847	0.806	NA
WBC	2, 207	34.50	<0.001	Primates > Carnivora > Ungulates

df = degrees of freedom; phylo.p = phylogenetic p value (see text); PSR = total parasite species richness; PSR_macro_ = macroparasite species richness; PSR_micro_ = microparasite species richness; WBC = mean white blood cell count;

* results remain qualitatively the same when the PSR is divided by citation count to control for differences in sampling effort.

In each of the models in Table 1, citation count (our measure of sampling effort) was highly significantly positively correlated with PSR. This confirms our suggestion that better studied mammals may appear to have more parasites than less well-studied species. If citation count is not included in the models, all groups except the ungulates show a significant positive association between longevity and PSR, although AIC values increase substantially (carnivores: longevity slope = 1.010±0.400, t_129_ = 2.741, p = 0.007, AIC = 460.5 [AIC with citation count = 367.2]; primates: longevity slope = 1.048±0.354, t_124_ = 2.958, p = 0.004, AIC = 384.3 [AIC with citation count = 325.7]; ungulates: longevity slope = −0.138±0.442, t_98_ = −0.313, p = 0.755, AIC = 369.2 [AIC with citation count = 345.4]). This would completely change our conclusions and thus highlights the importance of controlling for sampling effort in our models. Although citation count explains a great deal of the variation in PSR, models including citation count alone have much higher AIC values than our full models including body size and longevity (Table S4). Thus, our results are not completely driven by differences in sampling effort.

We did not find a significant positive relationship between host longevity and white blood cell counts (WBC; Table 3). However, we did find a significant negative association between WBC and PSR in ungulates using total PSR or microparasite PSR (Table 3). WBC was also significantly positively correlated with body mass across all three groups (Table 3).

**Table 3:** Phylogenetic generalized least squares models (PGLS) predicting mean white blood cell count (WBC) for Carnivora, Primates and terrestrial ungulates.

		Carnivora			Primates			Ungulates	
	**λ = 0.917**	**r^2^ = 0.169**	**AIC = −8.046**	**λ = 0.954**	**r^2^ = 0.072**	**AIC = −24.40**	**λ = 0.891**	**r^2^ = 0.259**	**AIC = −15.09**
**variable**	**slope**	**SE**	**t_59_**	**slope**	**SE**	**t_76_**	**slope**	**SE**	**t_69_**
PSR	−0.019	0.027	−0.718	0.025	0.023	1.119	−0.047	0.016	−2.837**
Longevity	0.000	0.097	−0.003	−0.035	0.106	−0.328	−0.032	0.069	−0.468
Body mass	0.083	0.029	2.894**	0.075	0.036	2.075*	0.084	0.026	3.264**
Citations	−0.001	0.029	−0.029	−0.017	0.019	−0.897	0.053	0.021	2.506*
	**λ = 0.847**	**r^2^ = 0.194**	**AIC = −8.844**	**λ = 0.959**	**r^2^ = 0.089**	**AIC = −25.76**	**λ = 0.907**	**r^2^ = 0.213**	**AIC = −11.02**
**variable**	**slope**	**SE**	**t_59_**	**slope**	**SE**	**t_76_**	**slope**	**SE**	**t_69_**
PSR_macro_	−0.041	0.031	−1.318	0.042	0.026	1.642	−0.038	0.020	−1.956
Longevity	0.050	0.103	0.485	−0.040	0.104	−0.386	−0.042	0.071	−0.596
Body mass	0.073	0.029	2.509*	0.075	0.036	2.073*	0.078	0.026	2.934**
Citations	0.014	0.031	0.458	−0.021	0.018	−1.140	0.046	0.022	2.111*
	**λ = 0.934**	**r^2^ = 0.166**	**AIC = −8.000**	**λ = 0.943**	**r^2^ = 0.057**	**AIC = −23.17**	**λ = 0.892**	**r^2^ = 0.248**	**AIC = −14.08**
**variable**	**slope**	**SE**	**t_59_**	**slope**	**SE**	**t_76_**	**slope**	**SE**	**t_69_**
PSR_micro_	−0.022	0.032	−0.682	−0.002	0.023	−0.068	−0.062	0.023	−2.643*
Longevity	−0.017	0.094	−0.176	−0.007	0.104	−0.066	−0.026	0.069	−0.373
Body mass	0.084	0.029	2.876**	0.075	0.036	2.059*	0.091	0.026	3.477***
Citations	−0.002	0.029	−0.061	−0.006	0.019	−0.297	0.057	0.022	2.548*

PSR  =  total parasite species richness; PSR_macro_  =  macroparasite species richness; PSR_micro_  =  microparasite species richness.

*** p<0.001;

** p<0.01;

* p<0.05.

## Discussion

Overall, our results show that there is, at best, a weak relationship between parasite species richness and longevity, at least in this dataset. Longer-lived ungulates have fewer parasites than short-lived species. However, analyses of primates and carnivores failed to produce significant associations between longevity and parasite burden, despite generally similar sample sizes. Several factors may account for the absence of a significant relationship between parasite species richness and longevity in these groups. Perhaps variables other than longevity are important in primates and carnivores; for example, Nunn et al. [13] found more compelling evidence for variables such as geographic range size predicting parasite species richness in primates. Other studies in carnivores also found no significant relationship between longevity and parasite burden [16]–[18]. Lindenfors *et al.* [18] suggested that this resulted from a limit to the number of parasites a host could acquire in its lifetime; if carnivores more quickly reach this saturation point, variation in longevity may have less influence on the number of parasites. Perhaps this is true in carnivores; however, it seems unlikely given that we found a significant relationship in ungulates, which, on average, live longer than carnivores. Alternatively, a negative relationship between longevity and parasite species richness in carnivores and primates may be counterbalanced by the loss of parasites as host longevity declines, as predicted by epidemiological theory. Indeed, the hypotheses are not mutually exclusive, and our tests will only detect a significant effect when one of the two hypotheses operates particularly strongly.

Epidemiological theory suggests that there should be a positive relationship between host longevity and parasite species richness [20]. Empirical evidence for such a positive correlation is, however, weak at best. We found no positive significant correlations between longevity and parasite species richness in our analyses, and although a few previous studies have found significant positive correlations in primates, Iberian carnivores and freshwater fish [13], [17], [21], two of these results only held when outliers were included [13] or body mass was excluded [21]. Thus, empirical evidence for the positive association between longevity and parasite species richness is generally lacking, suggesting that epidemiological processes involving mortality may have limited influence on the accumulation of parasite species in hosts [12].

If greater parasite burden generally favors low longevity in mammals, then when other ecological and social conditions favor high longevity, we might expect to find that animals invest in immune system defenses [55]. Thus, we should see a general association between longevity and investment in immune defenses, such as immune system cells circulating in the blood. However, we find no evidence for this hypothesis, with white blood cell counts showing no significant associations with longevity. This is in contrast to the results of Nunn *et al.* [40] who found positive correlations between longevity and monocyte and eosinophil counts in mammals (but only in females). One possible explanation for our results is that longer-lived mammals also invest more in behavioral anti-parasite defenses, for example, avoiding contaminated areas or individuals, allogrooming, or ingesting medicinal plants [9], [56]–[58]. Such defenses have been particularly well documented in social mammals like primates [58]. Equally, some long-lived species may simply not face high parasite risk due to their geographic location or ecology; thus, parasite infection may have little effect on their longevity. In addition, different parasites may select for different life histories. For example, chronic infections may select for increased immune investment and high longevity, whereas acute infections may select for decreased immune investment and faster reproduction. Our results did not detect any differences in response to macro- versus micro-parasites; however, these subdivisions may have imprecisely estimated the degree to which the parasites exhibit chronic versus acute effects.

Several methodological issues deserve mention. We used parasite species richness as a measure of parasite burden, but this ignores the intensity of infection: a host with one individual of each of 100 species of parasite may not be as negatively affected by parasites as a host with 1000 individuals of just one parasite species. The type of parasite involved may also matter; some parasites are more virulent than others and thus fitness costs will vary. Hosts should only invest in immune defenses if the cost of losses due to parasite infections exceeds the often high costs of immunity. Ideally intensity and virulence should be entered into our models. Finally, we only have data on three groups of mammals, all of which are fairly large-bodied and long-lived relative to the majority of mammals. It would be interesting to extend these analyses to include more species, particularly rodents and bats.

Our results indicate that longer-lived ungulates have fewer parasites than those that are short-lived, which supports previous studies in ungulates and other mammals [12], [14]. This effect may be caused by parasite-induced mortality, which would select for faster life-histories, rather than increased investment in anti-parasite defenses [12], [59]. These results may have implications for ungulate conservation. Generally long-lived mammals are at greater risk of extinction than short-lived species [60]. However, if the extinction driver involved is an emerging disease, short-lived ungulates may be hardest hit because they already harbor a greater parasite burden compared to long-lived ungulates, and also tend to exist at higher densities, which favors the establishment of infections. Short-lived ungulates are generally smaller and more abundant, and are therefore common prey items for large carnivores. Thus, if populations of short-lived ungulates experience a disease outbreak, it could have knock-on effects at higher trophic levels. This may also be the case in other taxonomic groups that were not part of our analysis. Further study of the links between parasite burden and host life-history are needed to allow us to protect biodiversity from infectious disease threats.

## Supporting Information

Supporting Information S1 Additional sources for life-history data.
(DOC)

Supporting Information S2 Dataset.
(XLSX)

Table S1 Models of parasite species richness including geographic range size and/or group size.
(DOCX)

Table S2 Full model predicting parasite species richness in Primates.
(DOCX)

Table S3 Phylogenetic signal in variables.
(DOCX)

Table S4 AIC values for full models vs. models containing only citation counts.
(DOCX)
