# Supplementary material for: Host Longevity and Parasite Species Richness in Mammals
Source: PLoS One. 2012 Aug 6;7(8):e42190. doi: 10.1371/journal.pone.0042190 (PMC3413396; doi:10.1371/journal.pone.0042190)
Supplement: Supporting Information S1 — Additional sources for life-history data. (DOC) [file pone.0042190.s001.doc]

**Appendix S1: Additional References**

Beauchamp, G., and G. Cabana. 1990. Group size variability in primates. Primates 31:171-182.

Bunker, A. 2007. "Canis simensis" (on-line), Animal Diversity Web. Accessed March 2, 2011 http://animaldiversity.ummz.umich.edu/site/accounts/information/Canis_simensis.html.

Encyclopedia of Life. Available from http://www.eol.org. Accessed 3 March 2011.

Gittleman, J. L. 1989. Carnivore behavior, ecology, and evolution. Ithaca, Cornell University Press.

Grzimek, B. 1990, Grzimek's encyclopedia of mammals. New York, McGraw-Hill.

King, S. J., S. J. Arrigo-Nelson, S. T. Pochron, G. M. Semprebon, L. R. Godfrey, P. C. Wright, and J. Jernvall. 2005. Dental senescence in a long-lived primate links infant survival to rainfall. Proc Natl Acad Sci USA 102:16579.

Lindenfors, P. 2002. Sexually antagonistic selection on primate size. J Evol Biol 15:595-607.

Primate Info Net: http://pin.primate.wisc.edu/index.html, Library and Information Service, National Primate Research Center, University of Wisconsin - Madison.

Nelson, A. P. W., and S. D. Williams. 2003. Grevy’s zebra survey: Kenya 2000. Final report. http://www.stlzoo.org/downloads/KenyaSurveyResults.pdf.

Nunn, C. L. 2002. A comparative study of leukocyte counts and disease risk in primates. Evolution 56:177-190.

Weigl, R. 2005, Longevity of mammals in captivity; from the living collections of the world. Stuttgart, Kleine Senckenberg-Reihe 48.
