# Supplementary material for: Host Longevity and Parasite Species Richness in Mammals
Source: PLoS One. 2012 Aug 6;7(8):e42190. doi: 10.1371/journal.pone.0042190 (PMC3413396; doi:10.1371/journal.pone.0042190)
Supplement: Table S1 — Models of parasite species richness including geographic range size and/or group size. (DOCX) [file pone.0042190.s003.docx]

**Table S1**: Phylogenetic generalized least squares models (PGLS) predicting total parasite species richness (PSR) for Carnivora, Primates and terrestrial ungulates.

|  |  | **Carnivora** |  |  | **Primates** |  |  | **Ungulates** |  |
| --- | --- | --- | --- | --- | --- | --- | --- | --- | --- |
|  | λ = 0.042 | r^2^ = 0.532 | AIC = 331.8 | λ < 0.001 | r^2^ = 0.430 | AIC = 344.1 | λ = 0.381 | r^2^ = 0.272 | AIC = 340.5 |
| **variable** | **slope** | **SE** | **t_110_** | **slope** | **SE** | **t_130_** | **slope** | **SE** | **t_95_** |
| Group size | 0.053 | 0.124 | 0.428 | 0.189 | 0.080 | 2.374* | 0.015 | 0.113 | 0.133 |
| Body mass | -0.118 | 0.064 | -1.854 | -0.024 | 0.064 | -0.377 | 0.002 | 0.125 | 0.015 |
| Citations | 0.785 | 0.072 | 10.85*** | 0.445 | 0.055 | 8.146*** | 0.499 | 0.088 | 5.646*** |
|  | λ = 0.036 | r^2^ = 0.506 | AIC = 334.2 | λ < 0.001 | r^2^ = 0.440 | AIC = 354.7 | λ = 0.268 | r^2^ = 0.272 | AIC = 356.1 |
| **variable** | **slope** | **SE** | **t_109_** | **slope** | **SE** | t_134_ | **slope** | **SE** | **t_100_** |
| GR size | 0.027 | 0.068 | 0.389 | 0.104 | 0.048 | 2.171* | 0.114 | 0.094 | 1.208 |
| Body mass | -0.118 | 0.070 | -1.682 | 0.049 | 0.056 | 0.868 | 0.008 | 0.116 | 0.072 |
| Citations | 0.744 | 0.076 | 9.746*** | 0.443 | 0.056 | 7.919*** | 0.446 | 0.094 | 4.736*** |

GR = geographic range; ***p < 0.001; *p < 0.05.
