# Supplementary material for: Host Longevity and Parasite Species Richness in Mammals
Source: PLoS One. 2012 Aug 6;7(8):e42190. doi: 10.1371/journal.pone.0042190 (PMC3413396; doi:10.1371/journal.pone.0042190)
Supplement: Table S2 — Full model predicting parasite species richness in Primates. (DOCX) [file pone.0042190.s004.docx]

**Table S2**: Phylogenetic generalized least squares models (PGLS) predicting total parasite species richness (PSR) for Primates. λ < 0.001; r^2^ = 0.474; AIC = 316.8.

| **variable** | **slope ± SE** | **t_110_** |
| --- | --- | --- |
| Longevity | -0.240 ± 0.307 | -0.780 |
| Group size | 0.144 ± 0.083 | 1.740 |
| GRsize | 0.098 ± 0.051 | 1.912 |
| Body mass | 0.023 ± 0.080 | 0.287 |
| Citation count | 0.462 ± 0.062 | 7.432*** |

GR = geographic range; ***p < 0.001.
