# Supplementary material for: Host Longevity and Parasite Species Richness in Mammals
Source: PLoS One. 2012 Aug 6;7(8):e42190. doi: 10.1371/journal.pone.0042190 (PMC3413396; doi:10.1371/journal.pone.0042190)
Supplement: Table S3 — Phylogenetic signal in variables. (DOCX) [file pone.0042190.s005.docx]

**Table S3**: Phylogenetic signal in the variables used in the analyses for Carnivora, Primates and terrestrial ungulates, estimated using Pagel’s λ (Pagel, 1999; Freckleton et al 2002).

|  | **Carnivora** | **Primates** | **Ungulates** |
| --- | --- | --- | --- |
| **variable** | **λ** | **λ** | **λ** |
| PSR | 0.032 | < 0.001 | < 0.001 |
| PSR_macro_ | 0.093 | 0.692 | < 0.001 |
| PSR_micro_ | 0.008 | 0.230 | < 0.001 |
| Longevity | 0.791 | 0.906 | 0.454 |
| Body mass | 0.992 | 1.000 | 0.974 |
| Citation count | 0.155 | 0.576 | < 0.001 |
| WBC | 0.948 | 0.953 | 0.942 |
