# Supplementary material for: Host Longevity and Parasite Species Richness in Mammals
Source: PLoS One. 2012 Aug 6;7(8):e42190. doi: 10.1371/journal.pone.0042190 (PMC3413396; doi:10.1371/journal.pone.0042190)
Supplement: Table S4 — AIC values for full models vs. models containing only citation counts. (DOCX) [file pone.0042190.s006.docx]

**Table S4:** AIC values from models containing all variables and models containing just citation counts, against various response variables for Carnivora, Primates and terrestrial ungulates. AIC values in bold indicate the model with the lowest AIC.

|  | **Carnivora** |  |  |  | **Primates** |  |  | **Ungulates** |  |
| --- | --- | --- | --- | --- | --- | --- | --- | --- | --- |
| **response** | **AIC_full_** | **AIC_citation_** | **AIC_difference_** | **AIC_full_** | **AIC_citation_** | **AIC_difference_** | **AIC_full_** | **AIC_citation_** | **AIC_difference_** |
| PSR_total | **367.2** | 405.7 | 38.5 | **330.0** | 357.5 | 27.5 | **345.4** | 353.5 | 8.10 |
| PSR_micro | **287.2** | 330.9 | 43.7 | **307.4** | 333.4 | 26.0 | **274.7** | 286.2 | 11.5 |
| PSR_macro | **353.9** | 392.8 | 38.9 | **307.8** | 329.2 | 21.4 | **326.5** | 333.0 | 6.50 |
| WBC | **-8.046** | -3.37 | 4.67 | **-24.4** | -26.7 | 2.30 | **-15.09** | -4.61 | 10.5 |
